# Teleexercise for Persons With Spinal Cord Injury: A Mixed-Methods Feasibility Case Series

**DOI:** 10.2196/rehab.5524

**Published:** 2016-07-14

**Authors:** Byron Lai, James Rimmer, Beth Barstow, Emil Jovanov, C Scott Bickel

**Affiliations:** ^1^School of Health ProfessionsUniversity of Alabama at BirminghamBirmingham, ALUnited States; ^2^Lakeshore FoundationBirmingham, ALUnited States; ^3^Electrical and Computer Engineering DeptUniversity of Alabama in HuntsvilleHuntsville, ALUnited States

**Keywords:** exercise, physical activity, telehealth, spinal cord injury, persons with disabilities

## Abstract

**Background:**

Spinal cord injury (SCI) results in significant loss of function below the level of injury, often leading to restricted participation in community exercise programs. To overcome commonly experienced barriers to these programs, innovations in technology hold promise for remotely delivering safe and effective bouts of exercise in the home.

**Objective:**

To test the feasibility of a remotely delivered home exercise program for individuals with SCI as determined by (1) implementation of the intervention in the home; (2) exploration of the potential intervention effects on aerobic fitness, physical activity behavior, and subjective well-being; and (3) acceptability of the program through participant self-report.

**Methods:**

Four adults with SCI (mean age 43.5 [SD 5.3] years; 3 males, 1 female; postinjury 25.8 [SD 4.3] years) completed a mixed-methods sequential design with two phases: an 8-week intervention followed by a 3-week nonintervention period. The intervention was a remotely delivered aerobic exercise training program (30-45 minutes, 3 times per week). Instrumentation included an upper body ergometer, tablet, physiological monitor, and custom application that delivered video feed to a remote trainer and monitored and recorded exercise data in real time. Implementation outcomes included adherence, rescheduled sessions, minutes of moderate exercise, and successful recording of exercise data. Pre/post-outcomes included aerobic capacity (VO_2_ peak), the Physical Activity Scale for Individuals with Physical Disabilities (PASIPD), the Satisfaction with Life Scale (SWLS), and the Quality of Life Index modified for spinal cord injury (QLI-SCI). Acceptability was determined by participant perceptions of the program features and impact, assessed via qualitative interview at the end of the nonintervention phase.

**Results:**

Participants completed all 24 intervention sessions with 100% adherence. Out of 96 scheduled training sessions for the four participants, only 8 (8%) were makeup sessions. The teleexercise system successfully recorded 85% of all exercise data. The exercise program was well tolerated by all participants. All participants described positive outcomes as a result of the intervention and stated that teleexercise circumvented commonly reported barriers to exercise participation. There were no reported adverse events and no dropouts.

**Conclusion:**

A teleexercise system can be a safe and feasible option to deliver home-based exercise for persons with SCI. Participants responded favorably to the intervention and valued teleexercise for its ability to overcome common barriers to exercise. Study results are promising but warrant further investigation in a larger sample.

## Introduction

In the United States, approximately 300,000 adults are currently living with a spinal cord injury (SCI) [1], and 50% of them report performing little to no physical activity other than their activities of daily living [2]. Those who report being physically active only engage in approximately 27 minutes of activity per week [3], a level substantially lower than the minimum recommended national guidelines for able-bodied adults [4] and recommendations made specifically for persons with SCI [5]. Because only a small percentage of persons with SCI are able to meet the national physical activity guidelines of 150 minutes per week of moderate aerobic exercise, it is not surprising that poor metabolic [6] and cardiovascular health [7] is often observed in this population. Additionally, those who are chronically inactive are at risk for secondary conditions including pressure ulcers, infections, and depression, which may even reduce life expectancy [8]. Such complications and deconditioning are preventable and often reversible by long-term, regular engagement in exercise. Unfortunately, persons with SCI have numerous barriers to exercise impeding their likelihood of adopting a consistent exercise routine [9].

The most commonly reported barriers to exercise by persons with SCI include both intrapersonal issues (eg, lack of energy, motivation, or knowledge) and those related to the built or organizational environment (eg, lack of accessible or affordable fitness facilities, equipment, and/or knowledgeable staff) [9-11]. In an effort to assist individuals in overcoming these barriers, recent innovations allow health care providers to deliver services to people in their homes through communication technologies (eg, smartphone or live video feed through the Internet), referred to as telehealth. Advantages of telehealth over usual care include greater cost-effectiveness, increased social support and access, better care, and higher quality of life [12]. With regard to individuals with SCI, telehealth has been proven to help in the management of pressure ulcers [13] and implementation of other strategies to promote healthy behaviors [14]. However, less is known about the potential of telehealth interventions that offer remotely delivered exercise training, a subset of telehealth called teleexercise.

Conceivably, persons with SCI could overcome both intrapersonal and environmental barriers through teleexercise. Technology can provide them with real-time monitoring of physiological data (eg, heart rate, respiratory rate) with instructions via live video feed from a remote fitness expert, enabling them to receive motivational support and potentially more accurate, safe, and effective doses of exercise. Thus, monitored teleexercise holds promise as a method of intervention that can address many of the most commonly reported barriers to exercise. To address the question of whether a monitored Web-based exercise intervention is feasible for individuals with SCI, this study assessed three core areas of feasibility [15] through the following aims: (1) test the implementation of delivering the intervention successfully at the home; (2) explore the potential effects of the intervention on aerobic fitness, physical activity, behavior, and subjective well-being; and (3) assess the acceptability of the program through participant self-report.

## Methods

### Study Design and Participants

**Figure 1:**
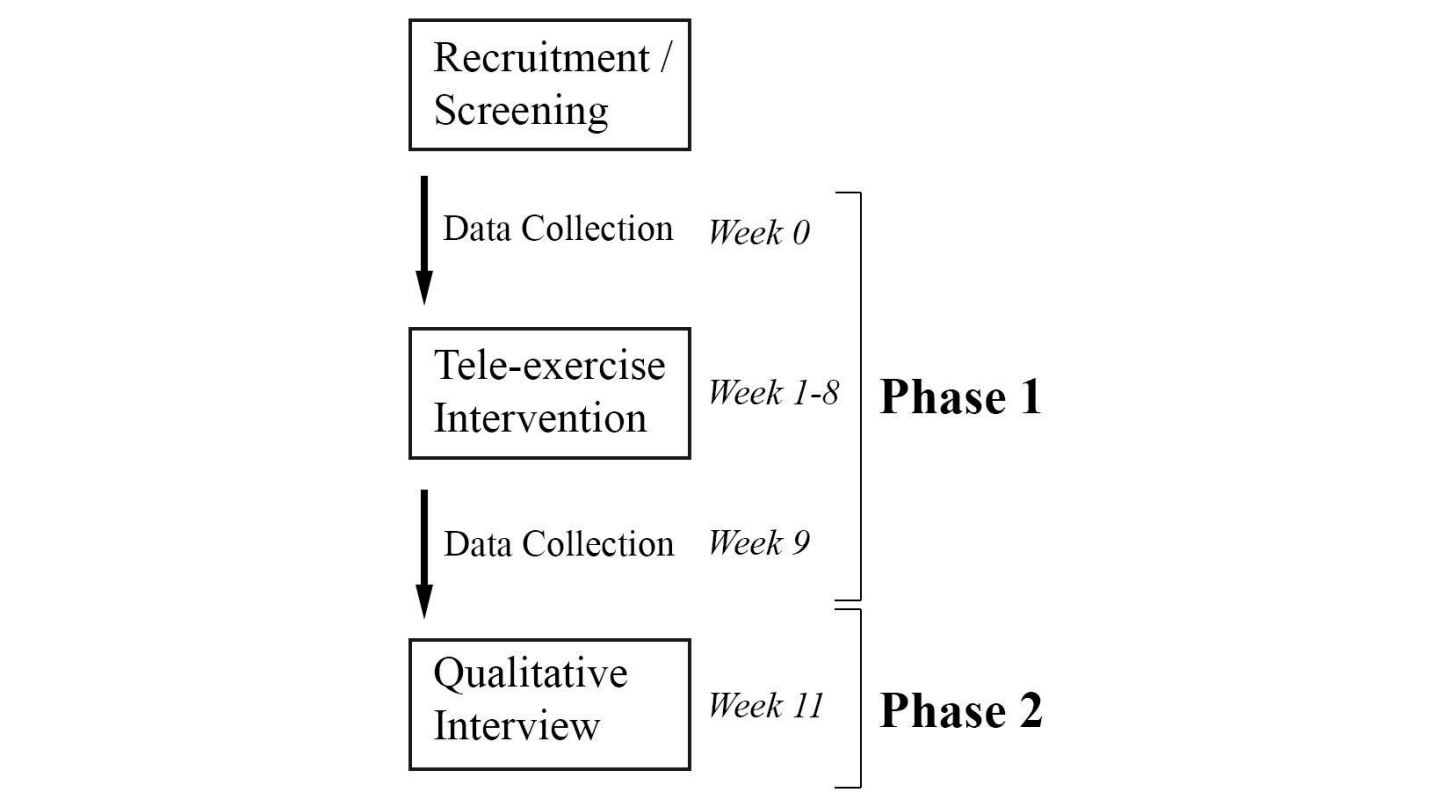
Study design and timeline: mixed-methods sequential design.

A convenience sample of four middle-aged adults (mean age 43.5 [SD 5.3] years; 3 males, 1 female; postinjury 25.8 [SD 4.3] years) with chronic SCI was recruited for a 2-phase (sequential) mixed-methods design [16] (Figure 1). Participant characteristics are shown in Table 1. The first phase, the intervention, consisted of 8 weeks of aerobic exercise with quantitative data collected pre- and postintervention. During the second phase, the intervention was withdrawn, and participants were instructed to resume their normal daily activities for 3 weeks. Participants were interviewed at the end of this period to qualitatively explore their perceptions of the program’s features and impact on their daily routine after completion. The arbitrary sample size of four was chosen to determine if the study could be administered as intended.

**Table 1:** Participant characteristics.

Participant	Age (years)	Sex	BMI^a^ (kg/m^2^)	Lesion level^b^	Years post injury
1	43	Female	19.5	T1^c^-T2	25
2	50	Male	27.1	T10-T11	28
3	44	Male	42.7	C4^d^-C5	30
4	37	Male	26.1	T2-T3	20

^a^BMI: body mass index.

^b^Lesion level: spinal cord injury level.

^c^T: thoracic.

^d^C: cervical.

Participants were eligible for inclusion in this study if they were aged 19 to 65 years and diagnosed with an SCI, used a wheelchair as their primary means of mobility, reported being physically inactive for 6 months prior to recruitment (no participation in a structured exercise program), were able to independently operate an arm ergometer; and had access to a wireless Internet connection. Participants were excluded if any known orthopedic, vascular, or cardiac problem interfered with the study protocol. This protocol was approved by the university’s institutional review board.

After participants provided written informed consent to the study protocol, they were instructed to come to the laboratory for pre- and postintervention data collection (week 0 and 9). During these visits, participant aerobic capacity (VO_2_ peak), quality of life, self-reported physical activity, satisfaction with life, and demographics were recorded.

### Intervention

#### Instrumentation

**Figure 2:**
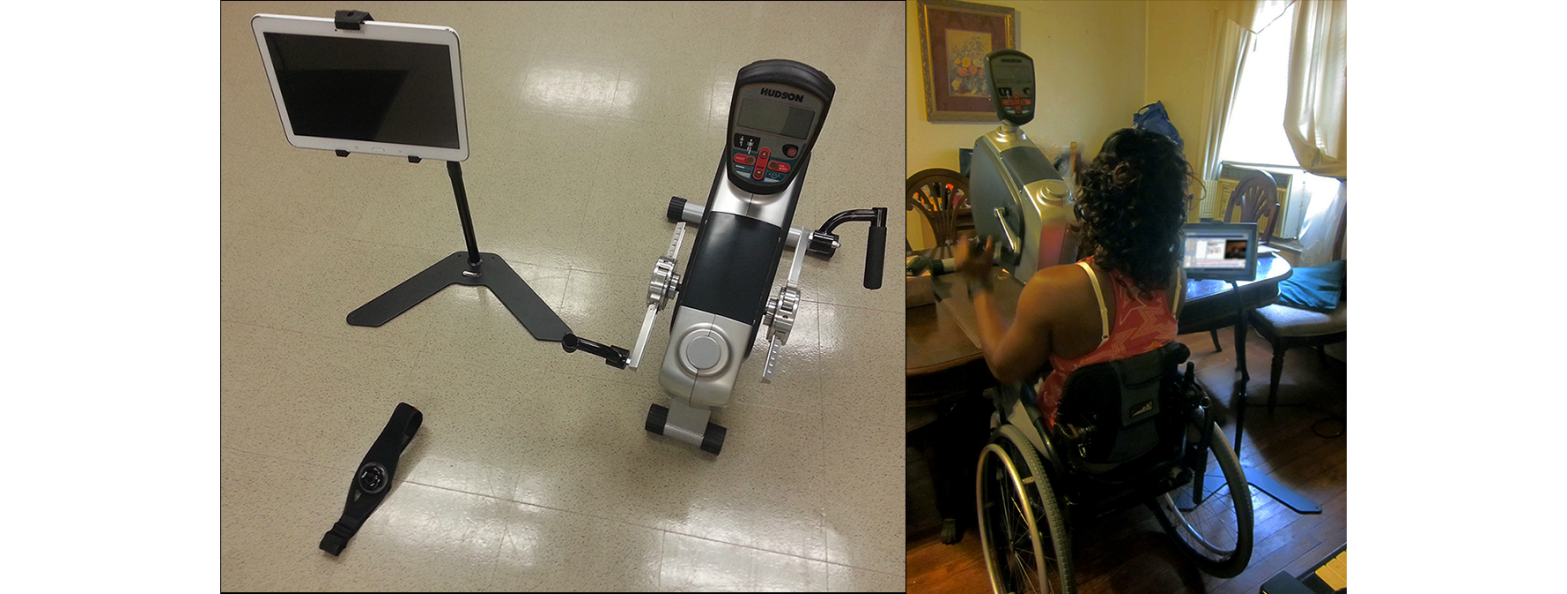
Equipment used in the intervention and a demonstration of the setup in the home.

**Figure 3:**
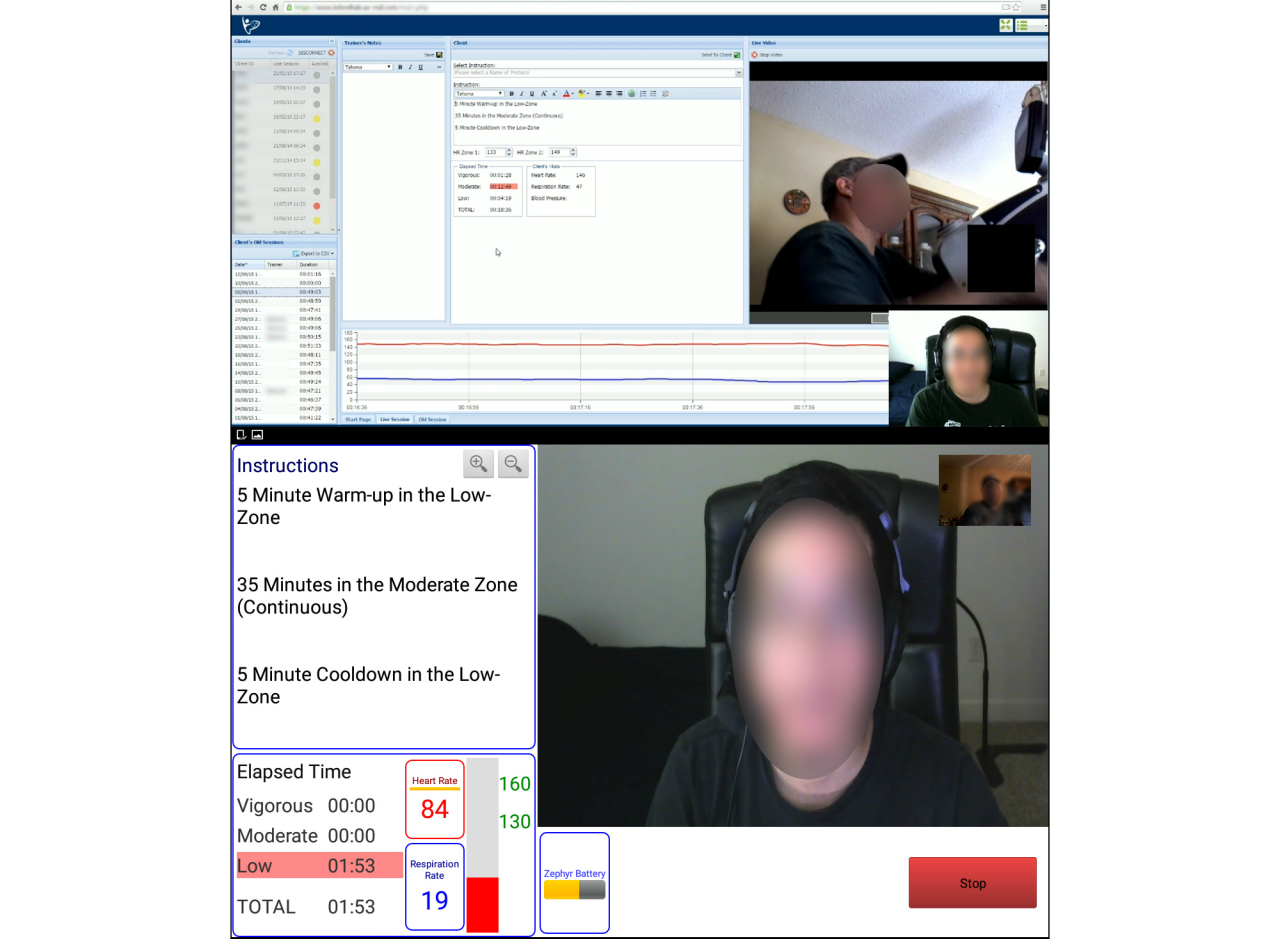
Exercise session from the telecoach's view (top) through online access to the dedicated server and the participant's view (bottom) from the custom-designed Web application.

The teleexercise intervention was delivered through a custom, wireless Internet-based system installed in the participant’s home. The equipment in this system included a tablet computer (Samsung Galaxy Tab 2 10.1, Samsung) with Bluetooth and wireless Internet capability mounted to an adjustable floor stand (Standzfree Universal Stand, Standzout); wearable physiologic monitor (Bioharness 3, Zephyr) that provided real-time monitoring of heart and respiration rate data to the tablet via Bluetooth connection; and custom-designed Web application that allowed physiologic data to be recorded from the tablet to a secure Web-based dedicated server. An example of this setup is shown in Figure 2. This platform allowed the exercise trainer (telecoach) to monitor each participant’s physiologic data in real time **(**up to 5-second delay) while simultaneously videoconferencing and providing written instructions to the participant. Written instructions served as an outline for daily and weekly exercise goals, which complemented verbal instructions given to the participant during the exercise session. For example, when asking participants to report their exertion level, telecoaches could provide a visual representation of a rating of perceived exertion (RPE) scale. The Web-based platform from the telecoach and participant perspective is shown in Figure 3. Telecoaches utilized this system to provide immediate feedback regarding exercise intensity and movement quality during each session. All exercise sessions were performed on an upper body ergometer (UBE-BDP Table Top Upperbody Exerciser, Hudson Fitness).

This study was designed to protect privacy and used state-of-the-art Internet data security mechanisms. First, no identifiable personal information was monitored or recorded through the teleexercise system. All personal information was stored separately on paper, and only the principal investigator had access. Second, the teleexercise system transferred all data, including physiologic and audiovisual communication, over a secured channel utilizing state-of-the-art encryption software. Physiological records were transferred to the remote server over HTTPS protocol based on 256-bit advanced encryption standard with cipher block chaining. Audiovisual communication between trainer and participant utilized WebRTC technology, based on peer-to-peer communication over Datagram Transport Layer Security protocol.

#### Intervention Protocol

The teleexercise intervention was delivered 3 times per week for 8 weeks (24 sessions). Sessions were separated by a minimum of 24 hours. Utilizing the teleexercise system, the training was delivered to the participants in their homes remotely by telecoaches located at the university research laboratory. To instruct and familiarize participants with the system, telecoaches conducted the first exercise session with each participant in the home after setting up the equipment. Additionally, telecoaches used this time to establish the regular exercise schedule with participants. Participants were allowed to choose the days and times they felt the exercise sessions would best fit their schedule. In the event participants could not attend or needed to reschedule an exercise session, they were informed to contact their telecoach via telephone. Participants were instructed to choose the day and time of the rescheduled session to avoid the telecoach influencing this variable. Lastly, they were told to report any injury or adverse event they experienced throughout the program to their telecoach.

During each exercise session, participants were instructed to maintain moderate exercise intensity, approximately 60% of their heart rate reserve (HRR **)** [17], using real-time heart rate data and collected RPE. The duration of each exercise session gradually progressed over the course of the 8 weeks with a goal of reaching 30 minutes of exercise (90 minutes total) at a moderate intensity by the fourth week of intervention. The 30 minute, 3 times-per-week exercise prescription was chosen to reflect the upper tier of aerobic exercise prescriptions commonly used in research for SCI [5,18]. The 4-week time frame was chosen based on a pilot test conducted prior to this study. At the start of the intervention, telecoaches set the goal of moderate exercise performed per session at a level that participants felt was comfortable. Each session included both a 5-minute warm-up and cool-down. Telecoaches then instructed participants to increase the duration of exercise when a participant could perform the moderate exercise minutes in two consecutive sessions and/or reported less than a moderate RPE (less than 3 on the modified Borg RPE 0-10 scale) [19] during moderate intensity exercise (indicated by heart rate data). Trainers encouraged participants to gradually increase duration of moderate exercise in increments of 5 to 10 minutes.

Telecoaches provided social support and assisted participants in maintaining moderate exercise intensity throughout the intervention. If a participant’s heart rate was too low during an exercise bout, telecoaches provided encouragement to increase the performed workload by either pedaling faster or increasing the resistance. Likewise, telecoaches strongly encouraged participants to lower their pace or resistance if participants exceeded the prescribed heart rate training zone. Telecoaches also monitored respiration rate for abnormalities in breathing. To avoid shoulder injury due to overuse, telecoaches instructed participants to alternate between forward and backward pedaling if severe muscle soreness occurred. Telecoaches prompted participants on a weekly basis to report any signs of injury or adverse events. To support the telecoach verbal instructions, exercise goals for each session (eg, a specific heart rate for a given amount of time) were provided in real-time written messages through the teleexercise platform. These messages provided participants with visual goals for the exercise session as a point of reference and an alternate means of communication in the case of audiovisual Internet lag. Lastly, telecoaches answered exercise-related questions raised by participants, but refrained from answering questions related to other lifestyle behaviors such as nutrition and diet.

### Outcome Measures

#### Implementation Outcomes

To assess the extent to which teleexercise can be successfully delivered in the home for persons with SCI, quantitative data including adherence, exercise session records, adverse events, and minutes of moderate exercise each week were recorded throughout the intervention.

Adherence to the intervention was defined as the percentage of total exercise sessions attended including rescheduled sessions. To be classified as a reschedule, the exercise session had to be performed before the next regularly scheduled session. If sessions were allocated to a later date past the next normally scheduled session, they were counted as a missed session (nonadherence). Based on previous studies [20], researchers considered 75% attendance to be considered acceptable.

To assess the stability of the monitoring technology of the Internet-based system, exercise recordings were assessed throughout the intervention. Successful exercise recordings were defined as the percentage of sessions that were monitored, recorded, and stored to a secure dedicated server over the Internet through the teleexercise Web application. A successful exercise recording required all data within these sessions to be saved successfully, including heart rate, respiration rate, and minutes of exercise. No published criteria for an acceptable percentage of exercise records have been established for this outcome.

Minutes of moderate exercise performed were recorded to evaluate the suitability of the intervention exercise prescription (ie, intensity and duration). Since the progression of the exercise prescription was exploratory in nature, no specific feasibility criteria were determined a priori. However, trainers aimed to guide participants toward the goal of 90 minutes of moderate exercise by the fourth intervention week. For exercise sessions where data were not able to be recorded through the teleexercise system due to technical difficulties (eg, Internet disconnection/disruption or equipment errors), minutes of moderate exercise were averaged for the remaining two exercise sessions performed that week.

#### Quantitative Outcome Measures

To provide future studies with an estimate of outcome variability for common health-related measures, quantitative outcomes included aerobic capacity and a set of health-related questionnaires that assessed the impact of the intervention on participant daily lifestyles.

Arm ergometers are generally held as an effective mode of aerobic exercise for persons with SCI [5,18]. Thus, peak oxygen consumption (VO_2_ peak, ml·kg^−1^·min^−1^), a gold-standard measurement of aerobic capacity, was assessed during a graded exercise test on an upper body ergometer. Prior to starting the test, participants were given a 3-minute rest period. Participants were instructed to maintain a pedaling cadence of 60 revolutions per minute while resistance was increased every minute by 10 watts until the participant reached volitional fatigue or achieved 3 of 5 criteria: age predicted heart rate max of more than 85%; RPE of 17 or more; respiratory energy exchange ratio of 1.1 or higher; plateau in oxygen consumption; or volitional fatigue [21]. Heart rate and oxygen consumption were recorded continuously during rest and exercise. Metabolic measures were taken using open circuit spirometry with a metabolic cart (TruOne, ParvoMedics). As a safety precaution, blood pressure was recorded before and after the exercise test. VO_2_ peak values reported for untrained male and female adults (young and middle-aged) with SCI (paraplegia) are defined as poor (less than 12 ml·kg^−1^·min^−1^), fair (12-15.3 ml·kg^−1^·min^−1^), average (15.3-17.7 ml·kg^−1^·min^−1^), good (17.7 -22.4 ml·kg^−1^·min^−1^), and excellent (more than 22.4 ml·kg^−1^·min^−1^) [22].

Since quality of life is closely linked to independent living, it has been identified as a critical outcome for therapeutic exercise [23]. In this study, quality of life was assessed by the Quality of Life Index [24] modified for SCI [25,26]. The QLI-SCI consists of 37 questions that assess importance and satisfaction with various aspects of life and utilizes a 6-point Likert scale from least satisfied/important to most satisfied/important. Questions are divided into 5 subscales: total quality of life, health and functioning, social and economic, psychological, and family. Scores from each subscale were combined into a total score using equations provided by the authors [27], with higher values representing a greater perceived quality of life. The general QLI has demonstrated excellent internal consistency (Cronbach alpha = .93) and test-retest reliability (*r*=0.87) and good validity with generic life satisfaction [24].

As an additional measure of subjective well-being **,** satisfaction with life was recorded using the Satisfaction with Life Scale (SWLS) [28]. The SWLS is a brief 5-question survey that utilizes a 7-point Likert scale from strongly disagree to strongly agree with scores ranging from 5 to 35. The SWLS has demonstrated good internal consistency (Cronbach alpha = .83) in persons with SCI [29] and good validity with other measures of well-being [30]. Higher scores indicate a greater degree of life satisfaction. Satisfaction with life has been identified as a common construct of well-being examined in exercise literature conducted for persons with SCI, with some evidence to suggest that it is positively affected by exercise [31].

To assess the influence of the exercise intervention on daily physical activity, physical activity was assessed using the Physical Activity Scale for Individuals with Physical Disabilities (PASIPD) recall questionnaire [32]. The PASIPD includes 13 questions related to the performance of activities of daily living over a 7-day period. End scores are converted into metabolic equivalents (MET hours/week). Scores can range from 0 (inactive) to more than 100 (very high activity). This instrument has demonstrated reliability and validity in a sample of persons with mobility impairment that included individuals with SCI [32,33].

#### Acceptability Outcomes (Qualitative)

Acceptability of the program was assessed qualitatively via participant self-report after program completion. Employing qualitative investigation in this manner has been suggested to enhance the overall content and depth of information provided by feasibility studies [34]. At week 11, 3 weeks after completion of the 8-week intervention, participants were interviewed. This time period was chosen to explore the possible impact of the intervention on participants’ daily routines and avoid reporting bias (social responsiveness), where participants provide answers at study completion they feel are in accordance with the expectations of the study or researchers, particularly when researchers view their outcomes [35]. The interview was semistructured, consisting of one ice-breaker question and 9 open-ended questions. These questions aimed to obtain participant feedback about the delivery of the teleexercise program, identify perceived advantages and disadvantages of the program, describe how their teleexercise experience might compare to a typical fitness facility, evaluate how the program affected their adherence, and explore the overall impact of the intervention from the pre-exercise baseline to the end of the 3-week follow-up period. An example of the interview questions and guide is provided in Multimedia Appendix 1. Participant interview data were recorded via audio devices and transcribed verbatim. Participants were given pseudonyms to ensure confidentiality of reported data. Interviews were conducted in a setting chosen by the participant (eg, the university research laboratory, their home).

### Analysis

#### Quantitative

Adherence was reported as a percentage of the prescribed exercise sessions attended during the intervention. VO_2_ peak and questionnaire data (quality of life, satisfaction with life, and 7-day physical activity recall) were reported at pre- and post-exercise intervention.

#### Qualitative

Two researchers analyzed qualitative data descriptively. The constant comparative method [36] was used to code emergent themes/categories from participant qualitative interview data. Within the constant comparative method, themes were coded and compared as they were collected for each participant. Within each participant’s interview data, events that emerged were first coded into initial categories or themes. After initial coding was completed, the emergent theoretical categories and their properties were reduced into fewer, more universal themes. The resultant major themes were reported. No statistical software was used. In the context of coding, analysts operated inductively within a post-positivism paradigm. In accordance with our objectives, this viewpoint was taken to focus coding on the participant perspectives and experiences, as opposed to a heavy interactive influence of the trainer (constructivist paradigm) [37]. Data were coded openly: no pre-existing criteria or themes were held.

Measures were taken to enhance the credibility and validation of the qualitative methodology. All interview data were transcribed by staff not involved with data analysis and reporting to prevent researchers from influencing the results to portray a certain outcome by recreating text, for example (experimenter bias). Additionally, qualitative data were checked by participants for accuracy (member checking) in two forms: (1) researchers asked participants to clarify ambiguous interview data and (2) themed data were cross-checked by participants for accuracy. Coding was first performed individually and then reviewed collectively by the lead investigator and a third-party reviewer, a method referred to as triangulation [38]. After individual codings were compared, researchers discussed their disagreements to resolve as many discrepencies as possible. This method, referred to as negotiated agreement [39], was employed to narrow the large variety of codes that could potentially be identified from open coding. Finally, for simplicity, interrater agreement among researchers was expressed as a proportionate percentage for major and minor themes [40]. The third-party reviewer had a background in qualitative research and had no direct involvement with the intervention, resulting in less intervention bias. The primary interviewer had a background in adapted physical activity and was a telecoach for the majority of the teleexercise sessions.

## Results

### Implementation Results

All four adults completed the intervention and were included in the final data analysis. Participants attended all 24 exercise sessions (100% adherence) with 8 of the total 96 sessions (8%) classified as reschedules. Reasons for rescheduled sessions included work-related conflicts (n=2), errands (n=2) out of town (n=1), Internet service provider issues (n=1), family obligations (n=1), and not feeling well (n=1).

Exercise sessions were successfully recorded to the dedicated server for 82 of the 96 sessions (85%) performed by the four participants. The primary causal factors for the 14 unsuccessfully recorded sessions were Internet connection/stability issues (9 occurrences) and irregularities in saved heart rate data (5 occurrences). One participant lived in an urban area and the other three participants lived in rural areas.

**Figure 4:**
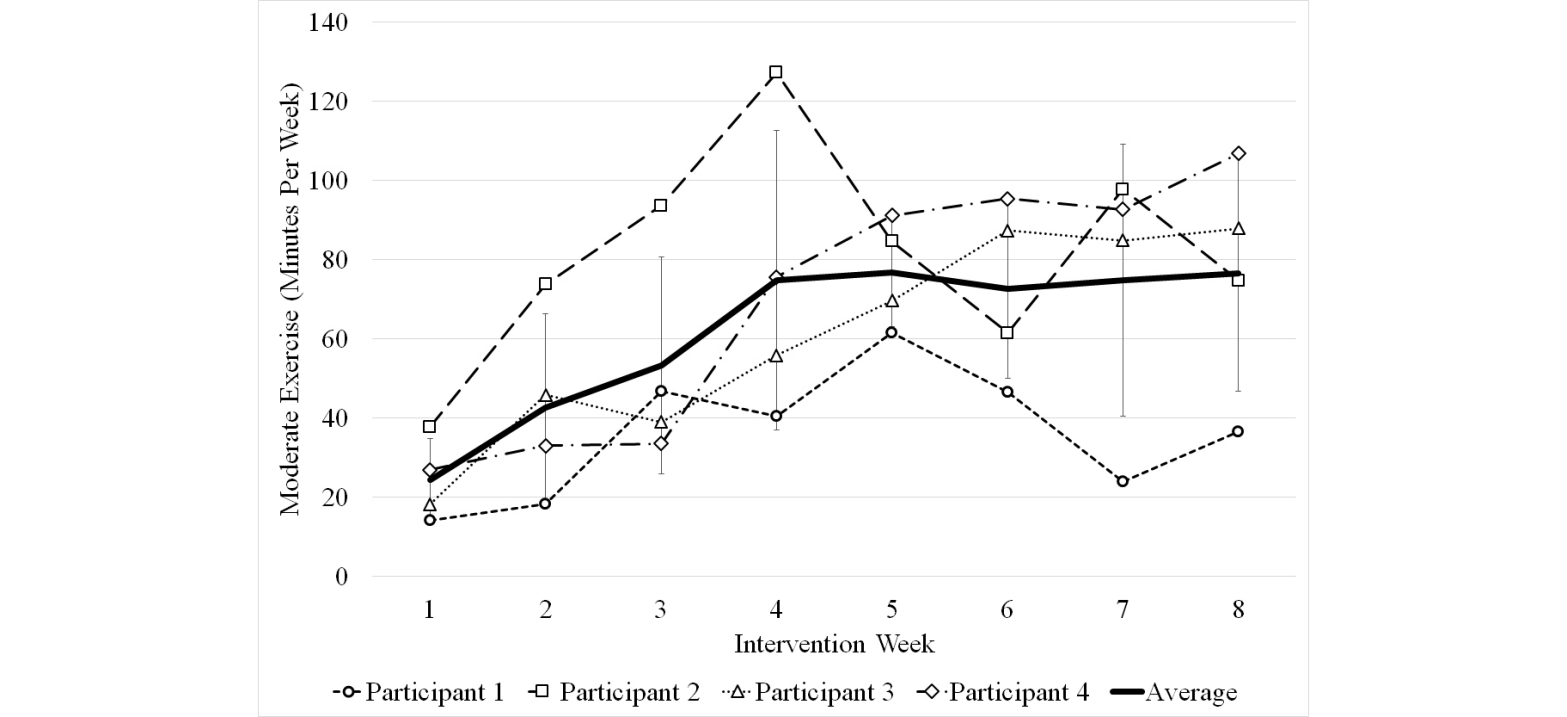
Minutes of moderate exercise performed per week.

Data were recorded in real time by the teleexercise system and categorized into either light/rest, moderate, or vigorous intensity exercise. Data for the four participants showed total minutes of exercise performed each week increased throughout the 8-week intervention (74.1 [SD 26.3] minutes at week 1 to 137.5 [SD 11.1] minutes at week 8). Participants appeared to plateau in the amount of moderate exercise minutes they achieved halfway through the intervention. Minutes of moderate aerobic exercise performed each intervention week are shown in Figure 4. At the start of the intervention (week 1) participants performed an average of 24.3 [SD 10.5] minutes of moderate exercise. At week 4, they achieved 74.8 [SD 37.8] minutes. At week 8, they held 76.5 [SD 29.7] minutes.

**Table 2:** Quality of Life Index: Spinal Cord Injury Version results.

Participant	Total score pre	Total score post	Health & function^a^ pre	Health & function post	Social & econ^b^ pre	Social & econ post	Psych^c^/spirit^d^ pre	Psych/spirit post	Fam^e^ pre	Fam post
1	23.5	22.2	24.1	23.7	23.1	22.6	21.7	21.7	24.3	19.2
2	17.1	18.5	13.9	16.9	20.1	20.2	23.8	22.6	10.9	10.3
3	18.5	22.0	17.8	21.7	18.1	23.3	18.6	23.5	19.1	17.9
4	22.7	20.7	23.4	21.1	22.9	20.4	22.7	21.4	18.0	16.6
Mean (SD)	20.5 (3.1)	20.9 (1.7)	19.8 (4.8)	20.9 (2.9)	21.1 (2.4)	21.6 (1.6)	21.7 (2.2)	22.3 (1.0)	18.1 (5.5)	16.0 (4.0)

^a^Functioning.

^b^Economic.

^c^Psychological.

^d^Spiritual.

^e^Family.

### Quantitative Outcome Measure Results

**Figure 5:**
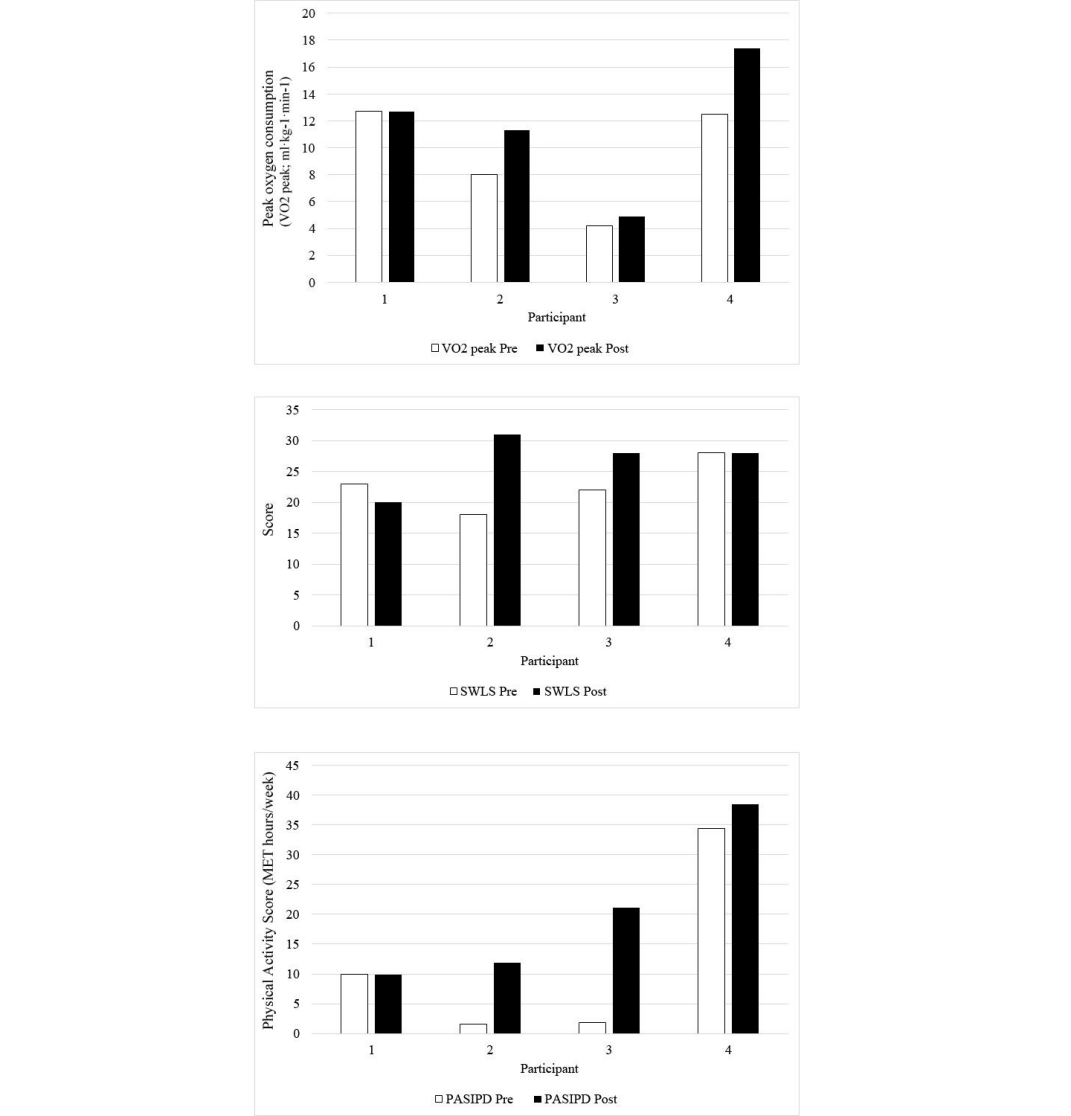
Peak oxygen consumption pre- and postintervention by participant; Satisfaction with Life Survey (SWLS) scores; reported physical activity performed over the past seven days (PASIPD).

Information for aerobic capacity, satisfaction with life, and physical activity data for each participant from pre- to post-data collection are shown in Figure 5. Responses varied among participants. The intervention appeared to have no impact on quantifiable outcomes for participant 1, who achieved the lowest amount of moderate exercise. Participants 2, 3, and 4 achieved a similar amount of moderate exercise and showed increases in VO_2_ peak values (ranging from 0.7 (18%) to 4.9 (39%) ml·kg^−1^·min^−1^) and daily physical activity (ranging from 4.13 to 19.3 MET hours per week), which likely implies the existence of a dose-training effect.

The two participants with the lowest aerobic capacity at the start of the study had the highest increases in daily activity and certain aspects of subjective well-being upon study completion. Participants 2 and 3, who reported the lowest MET hours per week and VO_2_ peak values at pre-data collection, showed increases of 10.3 and 19.3 MET hours per week, respectively. Additionally, they showed a 77% (from 18 to 31) and 27% (from 22 to 28) increase in SWLS scores, respectively. Likewise, in regard to quality of life, they showed increased scores in the health and function subcategory of the QLI-SCI (participant 2: pre=13.9, post=16.9; participant 3: pre=17.8, post=21.7). However, there did not appear to be any consistent notable differences overall in total or subscale scores on the QLI-SCI as shown in Table 2.

### Acceptability Results

Five major themes emerged from the qualitative interview data: (1) barriers to exercise at typical fitness facilities; (2) teleexercise as a solution to exercise barriers, (3) positive outcomes associated with teleexercise, (4) importance of the telecoach as a motivator, and (5) suitability of the employed teleexercise technology. Transcripts were independently coded by two researchers to ascertain emergent themes. Once transcripts were coded, the researchers met to discuss the analysis; interrater coder agreement was 100%.

#### Barriers to Exercise at Local Fitness Facilities

Participants identified numerous barriers to exercise at their local community fitness centers, including lack of access, convenience/time, usable equipment/program options, transportation, staff expertise in the area of disability, and high cost. Lack of transportation and convenience/time were noted by all four participants; access, usable equipment/program options, and staff expertise were identified by three participants.

I went to the gym. . . It’s probably not but five miles from the house. But there’s no accessible parking because they don’t expect people in wheelchairs to show up. And then I have to get into the gym itself. But then when you get into the door, there’s no way to even get around. I can go maybe ten or fifteen feet to get to some of the machines. . . I can’t even use them because their benches don’t come loose. . .

Participant 3

#### Teleexercise As a Solution to Exercise Barriers

Participants expressed a preference for teleexercise because they felt it provided a solution to exercise barriers, particularly those related to the environment. Specifically, all four participants acknowledged teleexercise as a convenient solution to exercise at a typical fitness facility. For example, participant 4 was employed full-time and also performed chores around his residence immediately upon arriving home from work. This participant performed his exercise sessions with a telecoach at 9 pm, a task he felt too difficult to do with an exercise trainer at a typical fitness facility which would require time allotted for transportation, transferring in and out of a wheelchair, and changing clothes. This participant successfully completed all 24 exercise sessions with only three of those sessions needing to be rescheduled.

I did it [teleexercise] more because it’s more convenient and on my time. You know I don’t have to make time to go there, get out of the truck, go in, and come back. You know you kill an hour easy. . . Well an hour and a half if you figure the time it takes to get out and go in, you know, get on your machine.

Participant 4

Three out of four participants identified teleexercise as an accessible and usable option versus going to a fitness facility.

A typical gym doesn’t even have the facilities for me to get a lot of the exercise machines. . . I could use free weights . . . but most of the machines were not adapted enough for me to use. . . There wasn’t really anything that I was doing that was aerobic.

Participant 2

It’s a step that I see as needed [teleexercise] because, as a quad, it is very hard to find exercise programs. I mean, the last exercise program that I had was in therapy while I was in the hospital as an inpatient. You don’t get the regimen of exercise as a quad because most gyms aren’t even slightly accessible.

Participant 3

#### Positive Outcomes Associated With Teleexercise

All four participants made several positive comments associated with the teleexercise program. These included increased energy/endurance and strength. They reported that these improvements increased their ability to perform physical activity. Additionally, three out of four participants mentioned that their increased physical capacity led to increased frequency and duration of physical activity and various occupations (meaningful, purposeful, and enjoyable forms of activity) after completing the intervention.

I think I’m 40% more active now since I’ve done it. . . I have a little more energy to go to the park. . . So, coming to the park and actually getting out and strolling around the park. . . I guess it has really gotten me out more.

Participant 1

The most impressive improvements in activity behavior were reported by those with the lowest physical capacity.

Before I would be up for about an hour, eat a meal, and then go to bed. This allowed me to stay up and interact and be a part of the family gathering. This was a really good side-effect of the program in that it built me up so I could stay up longer. . . I was stronger, had more mobility.

Participant 2

I can do what I did before (the intervention) but a lot more efficiently physically. So, I can get stuff done. Some things I can do faster. Some things I can do and still have energy. I can stay up and stay out longer. . . My days are 16 to 18 hours in the chair. Where I was at before was like 12 hours.

Participant 3

Participant 3 also described a noteworthy improvement in the amount of time spent participating in his physical activity/occupation. Participation in his weekly hobby, remote-controlled car racing within a community club, was impeded by a lack of energy prior to the intervention. The duration spent participating in his hobby with his friends increased from 1 to 2 hours to 4 to 7 hours after the intervention. He emphasized that this improvement enhanced his motivation to adhere to the teleexercise program.

I noticed after exercise that I could drive my car longer. Driving the car for me requires a lot of shoulder work because I have to hold my hands still while I’m controlling the car. . . Before the intervention I could race for ten to fifteen minutes then I’d have to take a break. But after the intervention, I could do it for an hour or two.

Participant 3

Participants 2 and 3 also reported sustained exercise behavior throughout the 3-week follow-up period after the intervention. During this period, both participants maintained and built upon the frequency and duration of their previous exercise regimens using arm cycles, which they had purchased via the Internet soon after the intervention was completed.

#### Telecoach As a Motivator

Participants appreciated the motivation and expertise that telecoaches provided through the teleexercise system. All four participants acknowledged the telecoaches as the primary facilitator of their motivation to adhere to the program. They acknowledged that the trainer provided monitoring, feedback, a social presence and bond, and gave them a sense of accountability to attend the exercise sessions.

I think it’s something that’s really useful as far as motivation. . . Having somebody checking in on me and asking about what I was doing and how I was doing. . . It made it go a lot faster in that you had somebody to talk to you while you were working out. . . I was accountable because someone was meeting with me.

Participant 2

Just having somebody there working out with you. You know that helps you, motivates you. Doing it by yourself you’re not going to push yourself as hard. You’ve got somebody there with you you’re gonna go harder, and plus it makes the time go by quicker when you’re sitting there talking with them.

Participant 4

#### Suitability of the Employed Teleexercise Technology

Participants acknowledged teleexercise technology as a feasible method for delivering exercise to a larger scale of persons with SCI but also noted several challenges. Three out of four participants identified issues with technology as a major disadvantage of teleexercise. One participant noted that the size of the tablet screen (10.5 inches) was challenging to read. Three participants noted Internet and tablet connectivity issues were interrupting and sometimes distracting with the exercise sessions.

The only issue I can think of would be of course the bandwidth. Bandwidth is a problem because you have to have a pretty solid upload and download speed.

Participant 2

In contrast, all four participants reported that the technology was easy to use.

I was familiar with the equipment, but I don’t think it was hard to use at all. Cause all you had to do was turn it on and click.

Participant 4

Most importantly, all four participants felt that teleexercise was capable of reaching a larger population of persons with SCI.

I just wish that more people that are. . . disabled, would participate in it. And it’s helpful, you know it’s like a starting point. . . For getting me up and out. You know, more active and motivated.

Participant 4

## Discussion

### Principal Findings

#### Summary

This study explored the feasibility of delivering a remotely monitored aerobic exercise program at home for persons with SCI. Overall, acceptable rates of adherence and recording and monitoring of exercise data suggest successful implementation of core intervention components. Encouraging preliminary findings from quantitative data included increased aerobic capacity, level of physical activity, and satisfaction with life, but these responses varied. In terms of acceptability, participants responded favorably to the intervention. They described positive outcomes as a result of the intervention. Furthermore, they described it as advantageous for overcoming barriers to exercise typically experienced at a fitness facility and identified their relationship with a telecoach as a critical component of their motivation to exercise. Taken together, this intervention provides fitness professionals with a preliminary model for delivering supervised exercise services to persons with SCI at home. Online fitness trainers are becoming more and more available but to our knowledge, there are no online personal training programs for persons with SCI.

#### Implementation

In regard to implementation, researchers felt the intervention was administered as intended. This was primarily suggested by the high rate of intervention attendance (100% vs the feasibility indicator of 75%) and no reported adverse events. Though 8% of sessions were rescheduled, researchers felt this rate was acceptable based upon their clinical experience with supervised exercise training. Additionally, researchers felt that successfully recording 85% of all exercise data was satisfactory considering the unpredictable nature of Internet stability and that all variables (heart rate, respiratory rate, and minutes of exercise) were required to be classified as a successful recording.

Of the exercise sessions that were not recordable, Internet disconnection issues were the primary causal factor. Initially, we attributed these issues to the fact that the intervention was primarily delivered in rural locations with frequent inclement weather conditions (ie, heavy rain and wind), both of which can affect Internet stability. However, the amount of disconnects decreased as telecoaches and research staff gained experience with the system; 86% (12/14) of unsaved exercise sessions occurred in sessions performed by the first two participants. Simple configurations, such as resetting or relocating the Internet router, greatly enhanced Internet stability. Difficulties experienced with Internet connectivity were similar to those reported in the literature [41,42]. Remote monitoring technology should aim to provide opportunities for exercise data to be saved after Internet disconnection and resumed once connection is restored. Additionally, telecoaches and/or research staff should implement mock training sessions to enhance familiarity with trouble-shooting various problems that can occur with the use of Internet technology in a home setting.

The exercise prescription required a more gradual progression than anticipated. The majority of participants in the present study were able to satisfy the minimum aerobic exercise guidelines for persons with SCI (40 minutes moderate exercise per week) [5], but they were far from reaching national aerobic exercise guidelines for adults established by the US Department of Health and Human Services [4] and the American College of Sports Medicine (150 minutes moderate exercise per week) [18]. Thus persons with SCI may require a longer progression of training to reach this target goal.

#### Potential Intervention Effects

Although our sample size limits statistical analyses, preliminary findings suggest the majority of participants experienced modest improvements in aerobic capacity and physical activity. Across the four participants, we observed a relative overall increase in aerobic capacity of 24%. As anticipated with exercise performed at a moderate intensity level [18], these gains are consistent to those reported by previous onsite aerobic interventions for SCI [43,44,45], and may also reflect increased satisfaction with life scores [46]. Quantitative findings appeared most prominent for those who performed a greater amount of moderate exercise or had lower starting values at the beginning of the study. In contrast, participant 1 (the only female) reported no improvements in quantitative data. It is unclear why some individuals respond more or less than others, which is the impetus for exercise dosing studies to inform more personalized exercise prescriptions. One potential explanation for this occurrence in participant 1 is that she performed a relatively lower weekly amount of moderate exercise compared to the other participants. In regard to quality of life, the duration of the current study was most likely too brief to achieve improvements observed in longer investigations [47]. Overall, these findings provide preliminary estimates of the variability of health-related exercise outcomes conducted for people with SCI. Further study is required to investigate these effects in a larger sample.

#### Acceptability

Participants provided positive feedback regarding physiological outcomes, the interaction with a telecoach, and the technology that was used in the teleexercise program. Although issues with Internet stability were described, all participants reported that the technology was easy to use. Participants noted that the technology removed several barriers to exercising at a local fitness facility, including not having to deal with inaccessible facilities and not demanding excessive amounts of time getting to and from the facility. These are common barriers to exercise for individuals with SCI [9-11]. Participants reported that the convenience of the program and the interaction with a telecoach contributed to their high adherence rates, suggesting that individuals with SCI can respond favorably to technology-based exercise programs at home.

### Future Directions

Several opportunities exist to enhance the technology used in the present study. First, future studies that aim to employ teleexercise should consider incorporating additional devices to enhance connection stability. For example, wireless access points can enhance stability in situations where computer tablets are located at great distance from an Internet router. Likewise, if Internet stability is the main concern, Ethernet adapters for computer tablets can allow direct Internet connection to a router and bypass issues with wireless Internet interference. In addition, future studies may benefit from incorporating innovative devices to enhance the visual clarity or overall user experience. One participant noted that the 10-inch screen tablet was challenging to read. Larger computer tablets or projection of data through digital cameras to larger digital screens, such as Smart TVs or computer monitors, may address this issue. Furthermore, trainers and research staff noticed participants often required assistance from a spouse/family member to equip heart rate monitors around their chest. Advances in wrist or upper arm heart rate monitoring technology will likely enhance the independence of teleexercise programs.

Qualitative findings indicate that one of the key benefits of the program as described by all participants was an increased physical capacity. These benefits allowed participants to engage in more healthy behaviors, particularly for those with lower baseline scores on physical capacity. It is unclear whether these benefits would be sustained over a longer time frame. Future studies that include the application of behavior change theories specific to physical activity are necessary to validate these findings. Specifically, these studies should examine strategies that can retain behavior over the long term (ie, 6 months to 1 year).

All participants valued the motivation and disability-related expertise provided by the telecoach, which they reported as a primary facilitator for attending the program. These findings are consistent with the theory of Support Accountability [48], a theory of behavior change developed specifically to account for the complex interaction of a health professional and consumer when communicating through electronic health technology. Under the lens of this theory, a person will be highly motivated to execute a healthy behavior if they know that a health professional, who they have a positive social relationship with, is waiting for them at a specific time through a technological medium. Although the inclusion of a trainer with remotely delivered electronic health technologies will heighten the costs of this program in a real world setting, supervised teleexercise might be ideal for people who lack sufficient motivation or knowledge to independently manage their own health through participation in exercise. Future studies may benefit from including behavior change theories that promote self-management of exercise behavior [49], which was beyond the scope of this study.

Since the primary aim of this study was to determine the feasibility of employing remote monitoring technology, this study used upper body arm ergometers, an established mode of aerobic exercise. However, the physiologic demand of these devices most likely contributed to the plateau in moderate exercise performed by participants, observed at approximately the fourth week of the intervention. Compared to traditional forms of aerobic exercise that utilize the lower limbs (eg, walking, jogging, cycling), arm ergometers rely on a relatively lower muscle mass in the upper arms, making participants more prone to early-onset fatigue [50]. Thus, to enhance training progression, as well as increase the effects of teleexercise on a wider variety of health-related outcomes, there is a need to identify exercise options that are effective in the home over a longer period of time targeting various types of activities for improving strength and cardiorespiratory fitness. Thus, future studies should pursue equipment that is cost effective and provides a variety of easily accessible and usable exercise options (eg, resistance bands, cuff weights, and adapted exercise equipment).

The demands on telecoaches were comparable to typical supervised exercise programs performed onsite, but the participants were much less burdened. The total demands on the telecoaches included virtually meeting with participants 3 days per week through the teleexercise system, two on-site visits to set up and withdraw the equipment, and the flexibility to reschedule an exercise session to a later date at the participant’s request. Participants appreciated the interaction and support they received from the telecoach. However, to improve sustainability in the community, promoters of teleexercise should develop strategies that potentially reduce the cost on the participant and/or time required by the telecoach. Such strategies could include increasing the participant-to-telecoach ratio (ie, group-based exercise) or tapering the amount of time spent with a telecoach throughout the study.

Teleexercise technology may serve as an adjunct to using fitness centers for promoting exercise in persons with SCI. Our findings suggest that the barriers of transportation, time to get to and from the exercise site, and inaccessible facilities prevent persons with SCI from engaging in regular exercise at local fitness centers. Teleexercise might address these issues by allowing fitness trainers to conveniently reach a wider variety of populations that desire supervised exercise training. Given that many fitness facilities often experience low volume during work hours (9 am to noon and 2 pm to 4 pm) there is potentially a 5-hour window for fitness professionals to serve as telecoaches and provide home exercise to people with disabilities for a nominal fee or as a small addition to their annual membership fee. Specific strategies for providing this online service warrant further investigation.

### Limitations

There were a few limitations in this study. First, the limited sample size prohibited statistical analysis. Second, participants might have been reluctant to express their negative opinions or criticisms of the teleexercise program to the researcher since the interviewer was a telecoach. Future studies should use independent evaluators to collect pre/post data who are not part of the telecoaching intervention. Lastly, exercise records and minutes of moderate exercise held no specific a priori criteria for feasibility.

### Conclusion

Persons with SCI experience substantial barriers to participating in community-based exercise. This Web-based intervention demonstrated good feasibility for remotely monitoring a moderate intensity exercise program for persons with SCI in the comfort of their home. Participants expressed high acceptability of the program, which they attributed to its accessibility, convenience, and the interpersonal interaction with the telecoach. Health professionals should consider expanding programs to include teleexercise for community-dwelling persons with SCI, especially among those living in rural areas who have limited or no access to onsite programs. The findings from this study are encouraging and merit further investigation in larger clinical trials.

## Multimedia Appendix 1

Example of the interview question guide used by the interviewer.

## Abbreviations

HRR: heart rate reserve
PASIPD: Physical Activity Scale for Individuals with Physical Disabilities
QLI-SCI: Quality of Life Index—SCI Version
RPE: rating of perceived exertion
SCI: spinal cord injury
SWLS: Satisfaction with Life Scale
