# Supplementary material for: Teleexercise for Persons With Spinal Cord Injury: A Mixed-Methods Feasibility Case Series
Source: JMIR Rehabil Assist Technol. 2016 Jul 14;3(2):e8. doi: 10.2196/rehab.5524 (PMC5454561; doi:10.2196/rehab.5524)
Supplement: Supplementary file 1 [file rehab_v3i2e8_app1.pdf]

Time of Interview:
Date:
Setting:
Participant Number:
Position of Interviewee:
Gender:
Age:
Does interviewee use a cane?
Does the interviewee use a walker?
Does the interviewee use a wheelchair? If so manual or power?
(Start with general to specific)

Introduction Part 1 (Interviewer Dialogue)

To explore your feedback about the tele-exercise program you participated in, you have been asked to participate in an interview after you complete the intervention. We are researchers from the X. The interview will contain 11 question that relate to your experience with the tele-exercise program. This interview should take less than an hour to complete. To clarify, tele-exercise is referred to as an internet-based exercise program delivered at the home. The interview will be recorded by audio and can be performed in a comfortable setting of your choosing. All information obtained from this interview will be strictly confidential. Only the research team will have access to this information. There are no inherent risks for participating in this interview. There are also no direct benefits for participating.

Do you agree or disagree to participate in the interview? (If Yes, then proceed to Part 2)

Introduction Part 2 (Interviewer Dialogue)

This study aims to investigate your (participant's) perceptions of the telexercise program, which you have recently completed. We will be using this information to publish a research study in the future. Just to clarify, we are defining telexercise as remote face-to-face training with a tele-coach with biofeedback, which is the real-time collection of physiologic variables: in this case this would be the heart and respiration data that was collected. This information is absolutely confidential, you will be given an ID#. Your name and any identifying information will not be collected. Before we begin, please try to be open and honest with your responses, try not to let my involvement with the project influence how you answer a question. So do you have any questions before we begin?

TExT-ME Interview Questions

(Probes for questions: "Can you explain what you mean"; "Tell me more about that")

1. What do you like to do on your free time (icebreaker question)
2. Tell me about your overall perceptions of participating in a teleexercise program performed at the home.
3. Describe to me some possible advantages of using a teleexercise program.
4. Describe to me some disadvantages or issues you experienced with using a teleexercise program.
5. Describe to me your thoughts about the face-to-face interaction with the telecoach/trainer.
6. Tell me your thoughts about the technology that was used in the teleexercise program.
7. How do you feel the teleexercise program will compare to going to a typical fitness gym?
8. Describe to me how you feel this program affected the amount of times you participated in the exercise sessions.
9. Describe your activity level prior to the intervention. As a prompt and follow-up. Tell me about activities you performed prior to the intervention.
10. Describe your activity level after the intervention. As a prompt and follow-up. Tell me about activities you now perform.
11. Do you have anything else that you would like to add?
